# Novel Lyssavirus in Bat, Spain

**DOI:** 10.3201/eid1905.121071

**Published:** 2013-05

**Authors:** Nidia Aréchiga Ceballos, Sonia Vázquez Morón, José M. Berciano, Olga Nicolás, Carolina Aznar López, Javier Juste, Cristina Rodríguez Nevado, Álvaro Aguilar Setién, Juan E. Echevarría

**Affiliations:** Centro Nacional de Microbiología, Madrid, Spain (N. Aréchiga Ceballos, S. Vázquez Morón, J.M. Berciano, C. Aznar López, C. Rodríguez Nevado, J E. Echevarría);; Centro Médico Nacional Siglo XXI, Mexico City, Mexico (N. Aréchiga Ceballos, Á. Aguilar Setién);; Centro de Recuperación de Fauna de Vallcalent, Catalonia, Spain (O. Nicolás);; Estación Biológica de Doñana, Andalucia, Spain (J. Juste);; Universidad de Alcalá de Henares, Madrid (C. Rodríguez Nevado);; Centro de Investigación Biomédica en Red de Epidemiología y Salud Pública, Madrid (S. Vázquez Morón, J.M. Berciano, C. Aznar López, J.E. Echevarría)

**Keywords:** Lyssavirus, Lleida bat lyssavirus, bent-winged bat, rabies, viruses, Spain

## Abstract

A new tentative lyssavirus, Lleida bat lyssavirus, was found in a bent-winged bat (*Miniopterus schreibersii*) in Spain. It does not belong to phylogroups I or II, and it seems to be more closely related to the West Causasian bat virus, and especially to the Ikoma lyssavirus.

Bats have been considered natural hosts of a wide diversity of viruses, including human pathogens such as lyssaviruses, severe acute respiratory syndrome coronavirus, henipavirus, and filoviruses (*1*). Within the genus *Lyssavirus*, 12 species have been described: *Rabies virus* (RABV), *Lagos bat virus* (LBV), *Mokola virus* (MOKV), *Duvenhage virus* (DUVV), *European bat lyssavirus* types 1 and 2 (EBLV-1 and -2), *Australian bat lyssavirus* (ABLV), *Aravan virus* (ARAV), *Khujand virus* (KHUV), *Irkut virus* (IRKV), *West Causasian bat virus* (WCBV), and *Shimoni bat virus* (SHIBV). Two more recently described viruses have not yet been classified: Bokeloh bat lyssavirus (BBLV) (*2*) and Ikoma lyssavirus (IKOV) (*3*). 

Bats are the natural reservoirs for most lyssaviruses, and to our knowledge, only MOKV and IKOV have never been detected in bats. RABV is the only virus known to establish epidemiologic cycles in bats and carnivores, and it is responsible for most human infections, mainly transmitted by dogs. The genus *Lyssavirus* comprises at least 2 phylogroups: phylogroup I (RABV, DUVV, EBLV1–2, ABLV, ARAV, IRKV, BBLV, KHUV) and phylogroup II (LBV, MOKV, and SHIBV). Phylogroup III consists of WCBV (*4*). According to a recent phylogenetic reconstruction that included the novel IKOV and was based on a fragment of 405 nt from the nucleoprotein gene, IKOV has proven to be highly divergent (*3*) and probably also forms part of phylogroup III.

During 1977–2011 in Europe, 988 cases of bat rabies were reported to the Rabies Bulletin Europe. Bats of the species *Eptesicus serotinus* and *E. isabellinus*, which account for >95% of cases, are considered the major natural reservoirs of EBLV-1. Several bat species within the genus *Myotis* are reservoirs for EBLV-2, BBLV, and the central Asian lyssaviruses ARAV and KHUV (*5*). WCBV has been isolated in the common bent-winged bat *Miniopterus schreibersii* (*6*). Other bat species might act as eventual hosts, although in Spain, bat rabies has been declared only in *E. isabellinus* bats (*7*). The possibility of a wider host range has been suggested by some surveys on natural bat colonies of other bat species describing neutralizing antibodies and genomic fragments related to EBLV-1 (*8*).

## The Study

In July 2011, a bat was found in the City of Lleida and taken to the Wildlife Care Center of Vallcalent (Lleida, Catalonia). The bat arrived lethargic and dehydrated, died soon after admission, and its carcass was frozen at −20°C. On March 12, 2012, as part of the rabies surveillance program in Spain, the bat carcass was received by the National Center of Microbiology, where rabies testing was conducted by 2 generic reverse transcription PCR (RT-PCR) methods for lyssavirus detection (*9*,*10*) and 2 commercial rabies antiserum assays (Bio-Rad Laboratories, Marnes La Coquette, France; and Fujirebio, Inc., Tokyo, Japan) for antigen detection by fluorescent antibody testing.

Brain smears were positive for lyssavirus by RT-PCR and fluorescent antibody testing, and an oropharyngeal swab sample was positive by RT-PCR. Further attempts to isolate the virus by tissue cultures were unsuccessful after 2 blind passages in BHK-21 and murine neuroblastoma cells. The negative results could be explained by the fact that the sample had been stored at −20°C for 8 months and had been frozen and thawed twice before cell culture testing; however, the possibility of the cell lines not being permissive for the virus cannot be excluded. 

The bat was morphologically identified as a bent-winged bat (*M. schreibersii*) and genetically identified by cytochrome b sequencing (*11*). The genomic sequence of the corresponding fragment of the diagnostic RT-PCR on the conserved region of the nucleoprotein gene, determined by BLAST (http://blast.ncbi.nlm.nih.gov/), showed no substantial sequence similarity to previously known lyssaviruses.

To determine the identity of the lyssavirus, we sequenced a larger fragment (565 bp), including the variable codifying region of the nucleoprotein gene (GenBank accession number submitted). We reconstructed an overall phylogeny of lyssaviruses by using a Bayesian Inference with the first 405 nt of the N-gene and MrBayes version 3.1.2 (http://mrbayes.csit.fsu.edu/). Two simultaneous runs of 10^6^ generations were conducted, each with 4 Markov chains, and trees were sampled every 100 generations. The best-fit nucleotide model, GTR + I + G, available in MrBayes was selected according to the corrected Akaike information criterion. The phylogenetic reconstruction was based on a dataset representative of all known lyssaviruses, including the recently described IKOV. The topology obtained showed that this sequence is more closely related to IKOV and WCBV than to the lyssaviruses in phylogroups I and II (Figure). These results suggest that this sequence tentatively belongs to a new *Lyssavirus* species named after the location of collection, Lleida bat lyssavirus (LLEBV).

**Figure:**
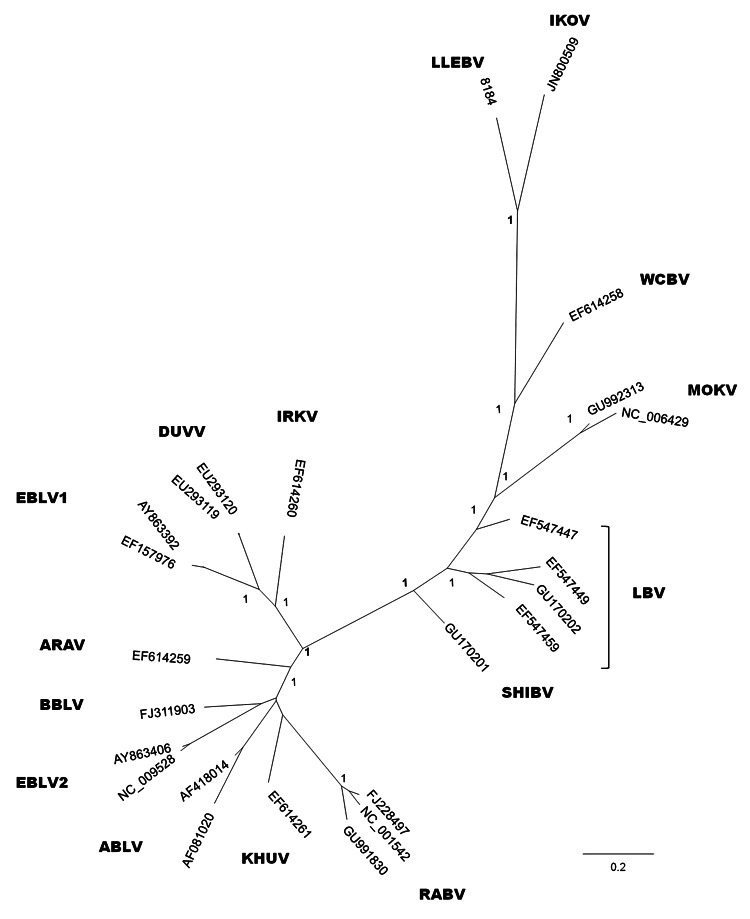
Phylogenetic reconstruction based on the first 405 nt of the nucleoprotein gene, including all representative lyssaviruses. The tree was obtained by Bayesian inference, and the first 25% of trees were excluded from the analysis as burn-in. Node numbers indicate posterior probabilities. ARAV, Aravan virus; ABLV, Australian bat lyssavirus; BBLV, Bokeloh bat lyssavirus; DUVV, Duvenhage virus; EBLV-1 and EBLV-2, European bat lyssavirus types 1 and 2; IRKV, Irkut virus; KHUV, Khujand virus; LBV, Lagos bat virus (lineages A, B, C, and D); MOKV, Mokola virus; RABV, rabies virus; SHIBV, Shimoni bat virus; WCBV, West Caucasian bat virus; IKOV, Ikoma lyssavirus; LLEBV, Lleida bat lyssavirus (proposed). Scale bar indicates expected number of substitutions per site.

The highest nucleotide identity was with IKOV (71.6%), followed by SHIBV (68.6%), IRKV (68.1%), KHUV (67.6%), EBLV-2 (67%–68.2%), ARAV (67.3%), WCBV (67.4%), ABLV (66.6%–67.7%), BBLV (66.1%), LBV (65.7%–68.6%), MOKV (65.7%–67.2%), DUVV (65.5%–65.8%), RABV (64.7–66.4%), and EBLV-1 (63.7%–64%). The lowest nucleotide identity was with the only lyssavirus found in bats of the Iberian Peninsula, EBLV-1. The nucleotide identity among the previously known lyssaviruses was 63.5%–80.0% in this particular fragment, and the lowest identities among strains belonging to the same lyssavirus were 80.4% for ABLV and 79.9% for LBV (the most distant LBV strain has been suggested to be a different lyssavirus) (Technical Appendix).

## Conclusions

The lyssavirus-specific antigen reactivity and association with a genomic sequence found in a bent-winged bat in northeastern Spain could be derived from the tentative new virus LLEBV. According to our phylogenetic reconstruction, the virus does not seem to belong to phylogroup I, which comprises most bat lyssaviruses, or to the African phylogroup II. The evolutionary relationships between the LLEBV sequence with WCBV and IKOV sequences need to be clarified before it can be determined whether they form >1 different phylogroups. 

Of note, the new LLEBV was detected in *M. schreibersii* bats*,* as was WCBV, the other European lyssavirus outside phylogroup I. The genus *Miniopterus* has traditionally been considered to belong to the family Vespertilionidae as do other bat genera linked to lyssaviruses in Eurasia (*Eptesicus, Myotis,* and *Murina*). However, recent molecular analyses have confirmed that the genus *Miniopterus* belongs to the family Miniopteridae (*12*). *M. schreibersii* bats are migratory, widely distributed across southern Europe and Eurasia. Large numbers (thousands) of these bats overwinter in caves and move in the spring to different and sometimes distant summer roosts for reproduction (*13*). These ecologic features make it relatively easy for an infectious agent to quickly spread out within and among the populations.

Consequently, it is difficult to imagine that WCBV or LLEBV are locally restricted; both could be located far from where they were found. Neutralizing antibodies against WCBV have been found in bats in Africa (*14*). The cumulative description of new bat lyssaviruses in recent years shows the convenience of always using generic amplification primers for rabies diagnosis based on RT-PCR to complement antigen detection.

No human exposure to the new virus has been reported. However, because of the divergence exhibited by LLEBV and IKOV, and the growing evidence of inadequate protection/cross-neutralization against viruses outside phylogroup I, the effectiveness of current rabies vaccines remains for these viruses a concern (*15*).

Technical Appendix Table of nucleotide identities (upper diagonal) and similarities (lower diagonal). Analysis was performed with the 405-nt fragment of the N-gene of all the known *Lyssavirus* species.

## References

[1] Calisher CH, Childs JE, Field HE, Holmes KV, Schountz T. Bats: important reservoir hosts of emerging viruses. Clin Microbiol Rev. 2006;19:531–45. 10.1128/CMR.00017-06 16847084 PMC1539106

[2] Freuling CM, Beer M, Conraths FJ, Finke S, Hoffmann B, Keller B, Novel lyssavirus in natterer’s bat, Germany. Emerg Infect Dis. 2011;17:1519–22 . 10.3201/eid1708.110201 21801640 PMC3381583

[3] Marston DA, Horton DL, Ngeleja C, Hampson K, McElhinney LM, Banyard AC, Ikoma lyssavirus, highly divergent novel lyssavirus in African civet. Emerg Infect Dis. 2012;18:664–7. 10.3201/eid1804.111553 22469151 PMC3309678

[4] Kuzmin IV, Wu X, Tordo N, Rupprecht CE. Complete genomes of Aravan, Khujand, Irkut and West Caucasian bat viruses, with special attention to the polymerase gene and non-coding regions. Virus Res. 2008;136:81–90. 10.1016/j.virusres.2008.04.021 18514350

[5] Schatz J, Fooks AR, McElhinney L, Horton D, Echevarria J, Vázquez-Moron S, Current state of bat rabies surveillance in Europe. Zoonoses Public Health. 2013;60:22–34. 10.1111/zph.12002 22963584

[6] Botvinkin AD, Poleschuk EM, Kuzmin IV, Bosisova TI, Gazaryan SV, Yager P, Novel lyssaviruses isolated from bats in Russia. Emerg Infect Dis. 2003;9:1623–5. 10.3201/eid0912.030374 14720408 PMC3034350

[7] Vázquez-Morón S, Juste J, Ibáñez C, Berciano JM, Echevarría JE. Phylogeny of European bat lyssavirus 1 in *Eptesicus isabellinus* bats in Spain. Emerg Infect Dis. 2011;17:520–3. 10.3201/eid1703.100894 21392449 PMC3166003

[8] Serra-Cobo J, Amengual B, Abellán C, Bourhy H. European bat lyssavirus infection in Spanish bat populations. Emerg Infect Dis. 2002;8:413–20. 10.3201/eid0804.010263 11971777 PMC2730232

[9] Echevarría JE, Avellón A, Juste J, Vera M, Ibáñez C. Screening of active lyssavirus infection in wild bat populations by viral RNA detection on oropharyngeal swabs. J Clin Microbiol. 2001;39:3678–83. 10.1128/JCM.39.10.3678-3683.2001 11574590 PMC88406

[10] Vázquez-Morón S, Avellón A, Echevarría JE. RT-PCR for detection of all seven genotypes of *Lyssavirus* genus. J Virol Methods. 2006;135:281–7. 10.1016/j.jviromet.2006.03.008 16713633

[11] Ibáñez C, García-Mudarra JL, Ruedi M, Stadelman B, Juste J. The Iberian contribution to cryptic diversity in European bats. Acta Chiropt. 2006;8:277–97. 10.3161/1733-5329(2006)8[277:TICTCD]2.0.CO;2

[12] Hoofer SR, Van der Bussche R. Molecular phylogenetics of the chiropteran family Vespertilionidae. Acta Chiropt. 2003;5:1–63. 10.3161/001.005.s101

[13] Serra-Cobo J, Sanz V, Martínez-Rica JP. Migratory movements of *Miniopterus schreibersii* in the north-east of Spain. Acta Theriol (Warsz). 1998;43:271–83.

[14] Kuzmin IV, Niezgoda M, Franka R, Agwanda B, Markotter W, Beagley JC, Possible emergence of West Caucasian bat virus in Africa. Emerg Infect Dis. 2008;14:1887–9. 10.3201/eid1412.080750 19046512 PMC2634633

[15] Both L, Banyard A, van Dolleweerd C, Horton D. Ma JKC, Fooks AR. Passive immunity in the prevention of rabies: a neglected tool for a neglected disease. Lancet Infect Dis. 2012;12:397–407. 10.1016/S1473-3099(11)70340-1 22541629

