# Supplementary material for: Novel Lyssavirus in Bat, Spain
Source: Emerg Infect Dis. 2013 May;19(5):793–5. doi: 10.3201/eid1905.121071 (PMC3647500; doi:10.3201/eid1905.121071)
Supplement: Technical Appendix — Table of nucleotide identities (upper diagonal) and similarities (lower diagonal). Analysis was performed with the 405-nt fragment of the N-gene of all the known Lyssavirus species. [file 12-1071-Techapp-s1.pdf]

Publisher: CDC; Journal: Emerging Infectious Diseases
Article Type: Dispatch; Volume: 19; Issue: 5; Year: 2013; Article ID: 12-1071
DOI: 10.3201/eid1905.121071; TOC Head: Dispatch

Novel Lyssavirus in Spain

http://dx.doi.org/10.3201/eid1905.121071

Technical Appendix

	1	2	3	4	5	6	7	8	9	10	11	12	13	14	15	16	17	18	19	20	21	22	23	24	25
1. LLEBV		71,6	68,6	68,1	67,6	68,2	67,0	67,3	67,4	66,6	67,7	65,7	65,9	67,2	68,6	65,7	67,2	66,1	65,8	65,5	65,4	64,7	66,3	63,7	64,0
2. JN80050 IKOV	71,6		63,7	67,4	66,2	65,4	65,4	64.5	69,5	65,3	64,6	66,0	64,0	68,1	64,9	63,5	64,7	66,0	65,0	64,5	64,0	64,2	63,8	65,3	66,0
3. GU170201 SHIBV	68,6	63,7		73,6	73,1	72,3	71,4	75,8	71,1	69,6	71,1	75,8	76,0	76,8	80,0	73,3	74,3	72,8	71,9	71,6	71,1	71,4	72,3	70,9	69,6
4. EF614260 IRKV	68,1	67,4	73,6		73,8	75,8	75,1	72.3	68,6	71,6	74,3	71,1	70,9	72,9	72,6	70,4	72,7	73,6	77,8	77,8	73,6	73,3	70,1	76,8	77,0
5. EF614261 KHUV	68,1	66,7	73,1	73,8		79,8	77,8	77,3	67,5	72,8	79,0	70,1	68,6	72,1	67,9	67,9	67,2	77,3	73,1	72,6	75,6	73,1	72,6	72,3	72,3
6. NC_009528 EBLV2	68,4	65,9	72,3	75,8	79,8		95,6	76,8	70,7	72,8	75,3	71,4	68,9	72,8	70,1	68,4	70,2	78,3	72,8	73,1	72,1	70,6	70,6	72,6	71,6
7. AY863406 EBLV2	67,2	65,7	71,4	75,1	77,8	95,6		76,5	70,4	74,1	75,8	72,3	69,9	73,8	68,9	68,2	68,2	80,0	73,6	73,8	74,1	70,9	70,1	74,8	73,8
8. EF614259 ARAV	67,7	64,7	75,8	72,3	77,3	76,8	76,5		70,6	73,6	76,8	73,6	72,8	73,8	72,3	72,1	73,1	77,3	73,3	73,1	71,9	70,9	73,6	75,3	75,6
9. EF61425 WCBV	67,4	69,6	71,1	68,6	67,7	70,9	70,6	70,6		68,1	69,7	72,8	74,1	71,9	74,1	69,4	71,1	70,7	70,1	69,9	67,2	66,9	66,9	68,1	67,9
10. AF081020 ABLV	66,9	65,9	69,6	71,6	72,8	72,8	74,1	73,6	68,1		84,4	71,9	72,1	72,1	71,1	72,3	70,4	74,8	72,8	73,3	72,6	73,6	72,6	72,8	73,1
11. AF418014 ABLV	67,9	64,9	71,1	74,3	79,0	75,3	75,8	76,8	69,9	84,4		71,1	74,1	72,1	69,6	72,8	72,6	79,8	76,3	75,8	76,0	74,8	74,8	72,6	72,6
12. EF547447 LBV	65,7	66,2	75,8	71,1	70,1	71,4	72,3	73,6	72,8	71,9	71,1		78,0	79,3	79,8	76,8	74,1	71,1	69,6	69,9	74,1	71,4	73,3	71,4	72,3
13. EF547449 LBV	65,9	64,2	76,0	70,9	68,6	68,9	69,9	72,8	74,1	72,1	74,1	78,0		81,2	81,2	75,8	73,6	72,8	73,1	72,6	70,1	70,1	71,9	71,4	70,9
14. GU170202 LBV	67,2	68,6	76,8	73,1	72,1	72,8	73,8	73,8	71,9	72,1	72,1	79,3	81,2		78,8	72,6	71,9	70,4	72,8	72. 1	71,4	72,3	73,1	71,4	72,3
15. EF547459 LBV	68,6	65,2	80,0	72,6	67,9	70,1	68,9	72.3	74,1	71,1	69,6	79,8	81,2	78,8		72,6	73,3	70,1	71,6	70,6	69,6	70,4	71,6	69,6	71,1
16. GU992313 MOKV	65,7	63,5	73,3	70,4	67,9	68,4	68,4	72.1	69,4	72,3	72,8	76,8	75,8	72,6	72,6		88,9	69,9	72,6	72,3	71,4	72,1	71,6	68,1	68,1
17. NC_006429 MOKV	67,2	64,7	74,3	72,8	67,2	70,4	68,4	73.1	71,1	70,4	72,6	74,1	73,6	71,9	73,3	88,9		69,6	72,4	71,9	70,2	70,5	69,9	68,5	68,7
18. JF311903 BBLV	66,4	66,7	72,8	73,6	77,3	78,3	80,0	77,3	70,9	74,8	79,8	71,1	72,8	70,4	70,1	69,9	69,6		72,6	72,3	75,1	72,3	73,1	74,1	73,6
19. EU293119 DUVV	65,9	65,2	71,9	77,8	73,1	72,8	73,6	73,3	70,1	72,8	76,3	69,6	73,1	72,8	71,6	72,6	72,6	72,6		99,0	72,6	70,9	70,6	78,8	78,0
20. EU293120 DUVV	65,7	64,7	71,6	77,8	72,6	73,1	73,8	73,1	69,9	73,3	75,8	69,9	72,6	72,1	70,6	72,3	72,1	72,3	99,0		73,1	70,9	71,1	78,0	77,3
21. FJ228497 RABV	65,4	64,0	71,1	73,6	75,6	72,1	74,1	71,9	67,2	72,6	76,0	74,1	70,1	71,4	69,6	71,4	70,4	75,1	72,6	73,1		92,3	86,2	72,1	71,6
22. NC_001542 RABV	64,7	64,2	71,4	73,3	73,1	70,6	70,9	70,9	66,9	73,6	74,8	71,4	70,1	72,3	70,4	72,1	70,9	72,3	70,9	70,9	92,3		85,4	70,9	69,6
23. GU991830 RABV	66,4	64,0	72,3	70,1	72,6	70,6	70,1	73,6	66,9	72,6	74,8	73,3	72,1	73,1	71,6	71,6	69,9	73,1	70,6	71,1	86,2	85,4		70,6	70,4
24. AY863392 EBLV1	63,7	65,4	70,9	76,8	72,3	72,6	74,8	75,3	68,1	72,8	72,6	71,4	71,4	71,4	69,6	68,1	68,6	74,1	78,8	78,0	72.1	70,9	70,6		95,1
25. EF157976 EBLV1	64,0	66,2	69,6	77,0	72,3	71,6	73,8	75,6	67,9	73,1	72,6	72,3	70,9	72,3	71,1	68,1	68,9	73,6	78,0	77,3	71,6	69,6	70,4	95,1	

Similarity
Identity
